# Promoting technical and methodological competencies in medical-oriented engineering degree courses by building digital transmitted-light and holographic microscopes: A pilot project

**DOI:** 10.3205/zma001782

**Published:** 2025-11-17

**Authors:** Christian Hanshans, Friederike Burkhardt

**Affiliations:** 1 Hochschule München, Fakultät VI, angewandte Naturwissenschaften und Mechatronik, Munich, Germany

**Keywords:** biomedical engineering, digital microscopy, microscopic anatomy, 3D printing, project-based learning, competency-based learning and assessment, open source

## Abstract

This work presents a structured cross-course teaching concept designed for biomedical engineering students, focusing on acquiring technical skills through the construction and application of 3D-printed robotic and holographic microscopes, spanning over different lectures. The project is embedded within the lectures of Medical Engineering, Medical Imaging and Anatomy and Physiology, aiming to bridge theoretical knowledge and hands-on applications.

The concept includes theoretical lectures, interactive seminars, and practical laboratory sessions as well as self-study phases and project implementation phases. Students are guided through the technical implementation such as 3D printing, wiring, programming and assembly as well as the use of the microscopes. Key learning objectives include physics, mastering manufacturing processes, and applying open-source resources and finally using their own built microscope within the practical part of a medical lecture. Besides that, the project-based approach should foster methodological competence and problem-solving skills as well as social competences and teamwork.

The first run shows increased student engagement and improved exam performance among participants, while qualitative feedback highlights the project’s motivational impact. Although designed for biomedical engineering students, the concept offers transferable elements for a wide range of usage within medical education. Besides the didactic aspects the use of DIY approaches based on open-source and 3D printing offer cost-effective, sustainable alternatives to cost-intensive, traditional lab equipment.

## Abstract (de)

Diese Arbeit beschreibt ein fachübergreifendes Lehrkonzept für medizinnahe Studiengänge. Die Studierenden bauen, testen und optimieren hierbei über ein Semester zwei Typen von Mikroskopen – kostengünstige, digitale, automatisierte Durchlichtmikroskope und 3D-holografische Mikroskope. Das Projekt ist in die Vorlesungen Medizintechnik, Medizinische Bildgebung sowie Anatomie und Physiologie eingebettet und zielt darauf ab, theoretisches Wissen mit praktischer Anwendung zu verknüpfen.

Das Konzept umfasst Vorlesungen, interaktive Seminare, Laborübungen und selbstständige Projektarbeit. Die Studierenden erlernen praktisch die technischen Schritte: 3D-Druck, Verkabelung, Programmierung, Zusammenbau und Anwendung der Mikroskope. Zu den wesentlichen Lernzielen zählen das physikalische Grundlagenwissen, die Anwendung von Fertigungsverfahren, die gezielte Nutzung von Open-Source-Ressourcen und schließlich der Einsatz des selbst konstruierten Mikroskops im praktischen Teil einer medizinischen Vorlesung. Darüber hinaus fördert der projektbasierte Ansatz methodische Kompetenzen, Problemlösefähigkeiten sowie soziale Kompetenzen und Teamfähigkeit.

Der erste Durchlauf zeigte eine gesteigerte Beteiligung der Studierenden und verbesserte Prüfungsergebnisse bei den teilnehmenden Studierenden. Qualitative Rückmeldungen hoben die motivationale Wirkung des Projekts hervor. Obwohl das Konzept für Studierende der Medizintechnik entwickelt wurde, enthält es gut übertragbare Elemente, die sich in vielen Bereichen der medizinischen Ausbildung nutzen lassen. Neben didaktischen Aspekten bieten Ansätze auf Basis von Open-Source und 3D-Druck kostengünstige und nachhaltige Alternativen zu teuren traditionellen Laborgeräten.

## Introduction

Biomedical engineering education faces persistent challenges in providing accessible, hands-on learning experiences that connect theoretical concepts with practical applications. Traditional lab equipment, especially high-end devices like robotic and holographic microscopes, is often prohibitively expensive, limiting its use in standard curricula. To address this gap, this project introduces an educational framework that enables students to design, construct, and apply DIY (do it yourself) microscopes using open-source technologies and affordable components.

The project is tailored specifically for engineering students in the field of biomedical engineering and is embedded in the courses “medical engineering 1” and “medical imaging” and “anatomy and physiology 1”. By integrating technical, methodological, and problem-solving skills, the project promotes a holistic learning approach aligned with socio-constructivist and situated learning principles. This hands-on initiative not only enhances technical competence in physics, engineering and rapid prototyping or programming but also strengthens soft skills like teamwork and project management.

This report focuses on the implementation and outcomes of this project, highlighting its potential as a transferable model for other cross-course and interdisciplinary educational settings within, biomedical engineering or medical education. While the primary aim was to advance technical learning objectives, the project also demonstrates how practical, project-based methods can foster motivation and engagement in anatomy and physiology courses, paving the way for interprofessional collaboration in clinical and biomedical contexts. Research supports the value of additive manufacturing in educational settings, demonstrating its potential to enhance student engagement and deepen understanding of complex concepts. In medical engineering as well in medicine, 3D printing has proven especially useful, enabling the creation of anatomical models, prosthetics, and other biomedical devices that facilitate experiential learning. Studies highlight the benefits of using 3D printing in fostering interdisciplinary collaboration and practical problem-solving skills among students [1]. Building on this foundation, this project implemented a DIY approach allowing students to construct and operate robotic or holographic microscopes. The curriculum of the studied cohort emphasizes active learning and skill building through hands-on experience [2], [3]. Moreover, the course design makes use of socio-constructivist principles, emphasizing collaborative problem-solving through group projects. This is intended to promote student involvement and knowledge exchange, thereby enhancing motivation and engagement in the learning process [4]. Research shows that situated learning enables learners to gain not only factual knowledge but also the tacit skills necessary for effective problem-solving and the development of professional competencies [5]. Blooms Taxonomy emphasizes the development of critical thinking and problem-solving skills, as students are required to perform higher-order thinking tasks that are directly linked to the learning objectives [6], [7], [8]. By ensuring that assessments are directly aligned with learning outcomes, educators can more accurately measure student understanding and competencies.

## Project description

**Figure 1:**
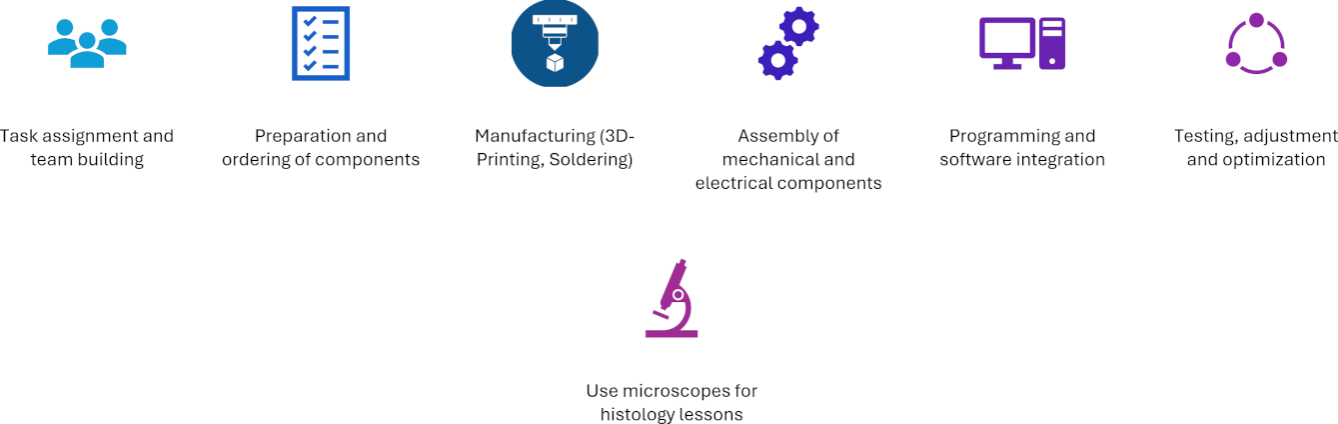
The project followed a structured timeline. These phases were supported by regular project meetings where progress and challenges were reviewed

In this project-based education model, students design and build digital robotic and 3D holographic microscopes using open-source assets, which they then use to learn about histology. In the process, they develop essential design, prototyping and application skills (see figure 1 (Fig. 1)).

**Figure 2:**
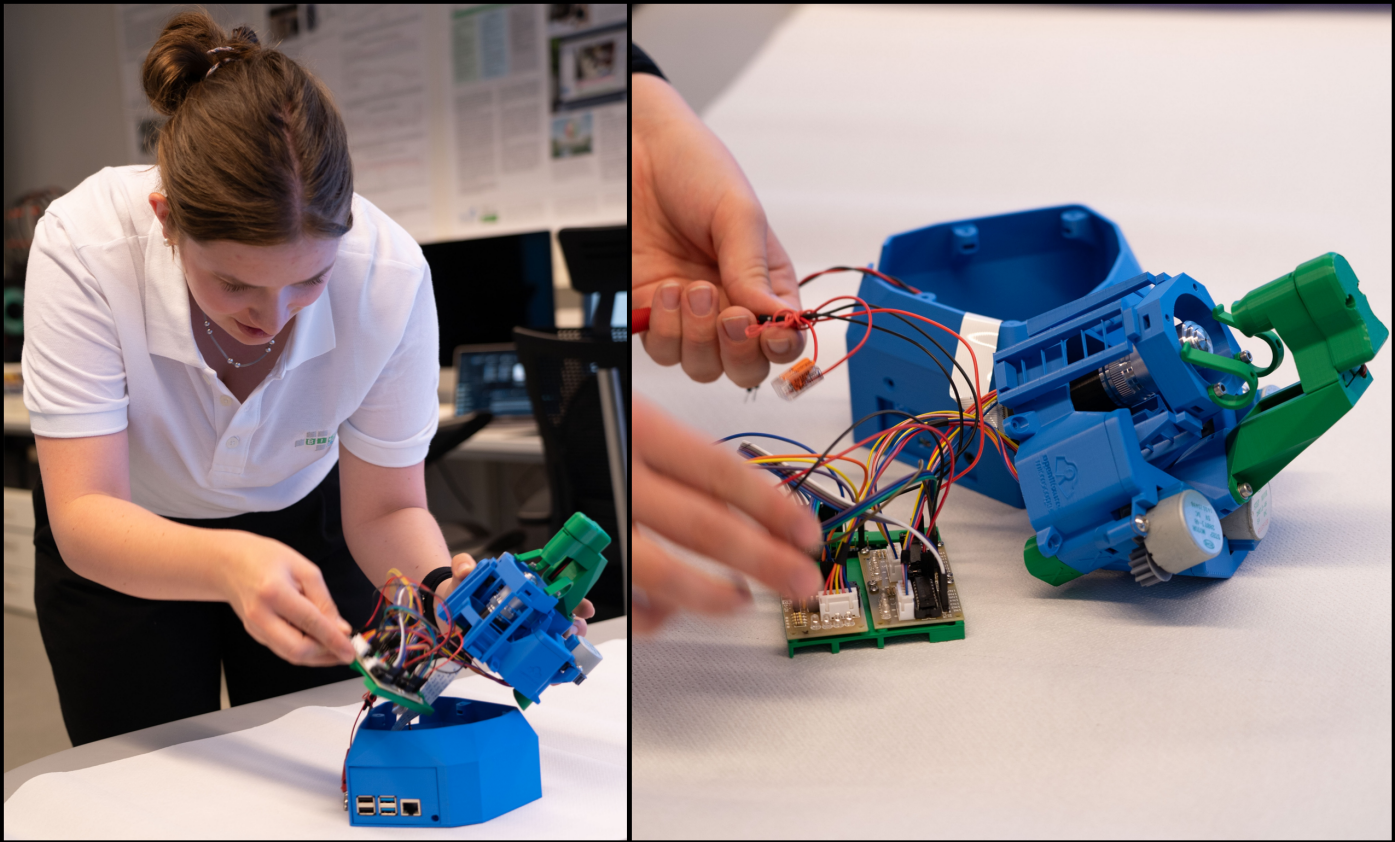
A student assembling the microscope (left) and organizing the hardware inside (right)

The build microscopes are based on open-source designs [3], [9]. Students were tasked with selecting appropriate materials based on mechanical or optical properties. They 3D-printed necessary parts and assembled the microscopes, incorporating electronic components and software (see figure 2 (Fig. 2)). The assembly and testing process involved iterative refinement, with students adjusting optics, optimizing mechanical fits, and testing overall functionality or adapting the software.

**Figure 3:**
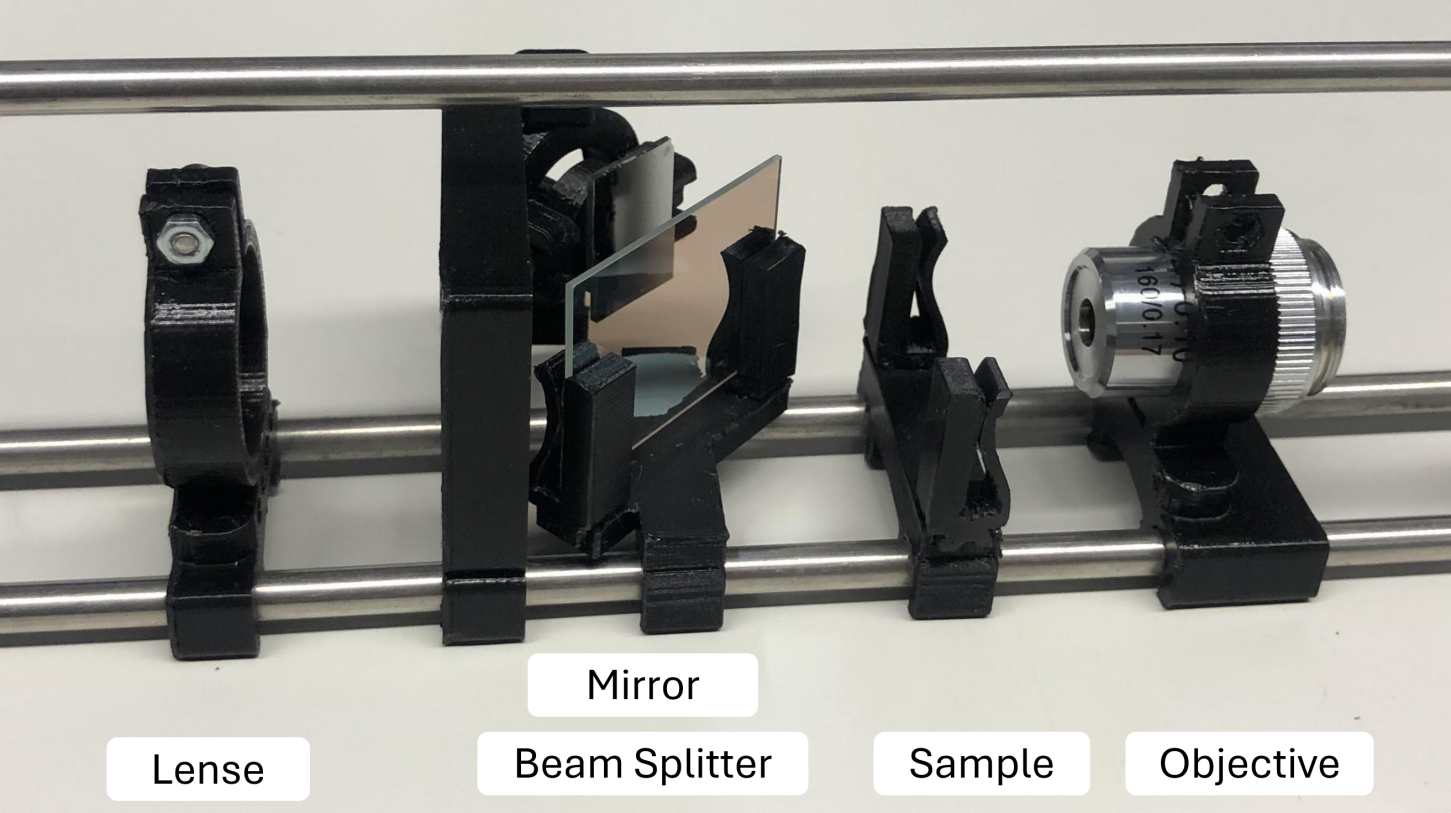
The holographic microscope is a highly specialized and complex device, allowing the observation of living cells without the need for staining

Building a robotic microscope is intended to improve mechatronic skills, as it focuses on mechanical construction and the integration of hardware and software systems. Students engage with topics such as actuation, sensors, and control systems. In contrast, the holographic microscope involves a relatively simple physical setup (see figure 3 (Fig. 3)) but presents more complex challenges in the areas of algorithmic image reconstruction, digital signal processing, and programming. These two options offer self-directed and individualized learning paths within the project.

A total of 19 Biomedical Engineering students (5^th^ semester) participated, working in teams over the whole semester. The project was continuously supported by a peer-teacher and technical staff with weekly sessions spanning theoretical lectures, interactive seminars, and practical lab sessions held by the professor. In the lectures, students were introduced to the fundamentals of physics, optical sensors, rapid prototyping techniques within the two technical classes as well as microscopic anatomy and microscopic applications within the medical class. Seminars allowed students to engage in discussions with peers and instructors, reinforcing the theoretical content and encouraging critical thinking. These sessions were structured to accommodate students with varying levels of prior knowledge, ensuring that all participants could effectively engage with the course material and collaboratively address challenges encountered during the practical sessions. 

Challenges and troubleshooting were integral to the learning experience. This initiative aims not only to strengthen technical and methodological skills but also to promote active problem solving and teamwork.

Following Bloom’s Taxonomy of learning, students are intended to acquire skills and competencies to foster both foundational knowledge and higher-order thinking. For example, on the “remember and understand” level, students were expected to recall microscopic structures of tissues, understand basic principles of rapid prototyping, as well as functions of microscopic techniques. Students “applied” this knowledge by 3D-printing components and programming microscope control units. They independently evaluated (“analyze”) optical quality and component compatibility. To “evaluate”, students critically assessed the performance of their devices against commercial microscopes. Finally, the “create” level was reached when teams designed and constructed functional microscopes for specific applications.

A blended assessment approach, combining traditional exams with project-based assessments, was used for this pilot project to determine students’ theoretical knowledge and practical skills [10]. Performance was rated by peer feedback, instructor assessment and the quality of the completed microscopes.

## Results

### Participation and engagement

Participation in the project was strong, with 69% of the students enrolled in the course (compared to the typical 40%). 95% (typically 50% within the study program) of the students who regularly attended the lecture, joined the voluntary semester project. 

Qualitative feedback from students highlighted the project’s motivational effect, with many expressing a sense of ownership over their work and an appreciation for the opportunity to work independently. One student voiced: “Building a microscope from scratch allowed me to understand the critical properties of each component and their contributions to the overall device functionality.”

Students reported that building their own microscopes helped them understand the critical components and their functional interdependencies. Feedback emphasized that the project fostered problem-solving skills, collaborative teamwork, and confidence in technical tasks such as rapid prototyping, soldering, and programming. In the parallel anatomy and physiology class, it was found that the students who had built the microscope performed 22% better in the lab courses about microscopic anatomy.

**Figure 4:**
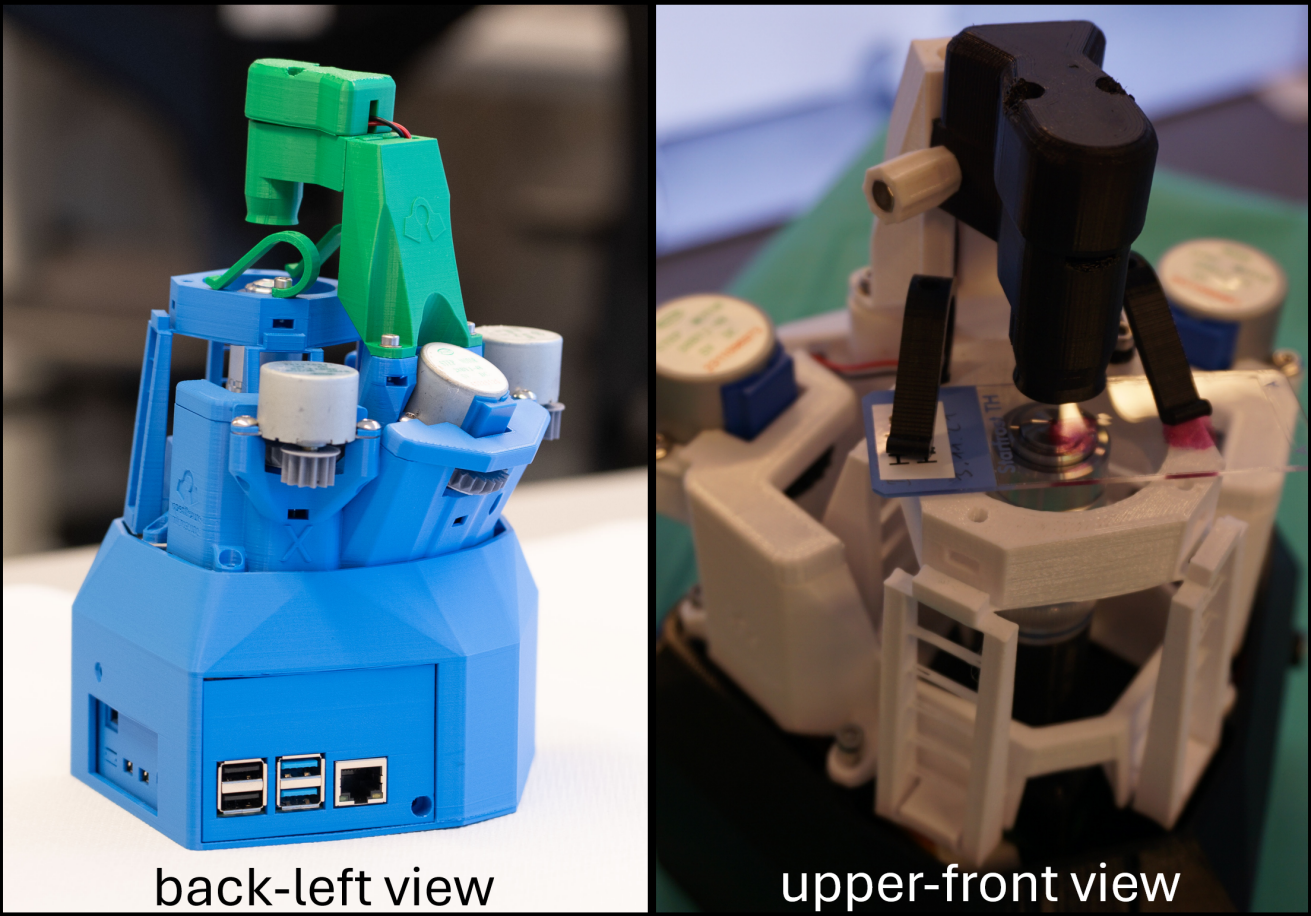
Student build 3-axis motorized brightfield microscopes

The self-constructed robotic (see figure 4 (Fig. 4)) and holographic microscopes were found to be comparable in quality to commercial devices, such as the Zeiss Axiolab 5 available in the lab (at 400x magnification). Additionally, the robotic microscopes enabled automated scanning of samples, offering a cost-effective and sustainable alternative to traditional lab equipment. 

**Figure 5:**
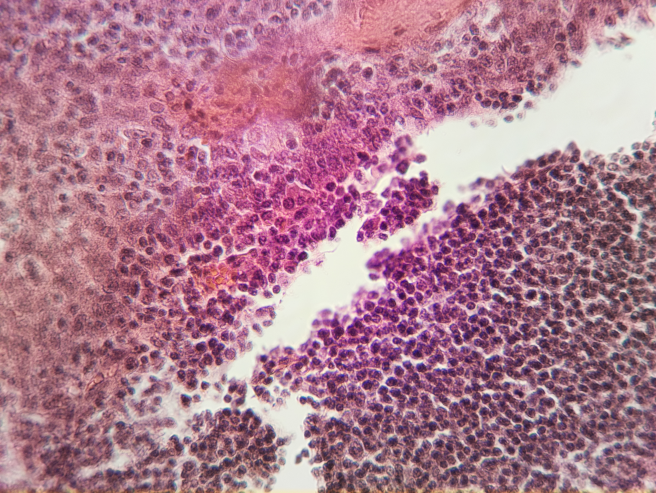
Example image of a human tonsil (HE stain) at 400x magnification taken with a student-built robotic microscope

Because the microscopes operate without an eyepiece objective, the image can be projected on a screen in real time. This allows all participants of each team of the course (each team typically of consists of 3 students) to observe a sample simultaneously while the instructor explains. This also led to vivid discussions within the team, that now was able to point at certain structures on the screen, whereas in former semesters (using traditional microscopes) discussions were less frequent and substantial. An example image of a student-build robotic microscope at 400x magnification showing lymphatic tissue can be seen in figure 5 (Fig. 5). 

**Figure 6:**
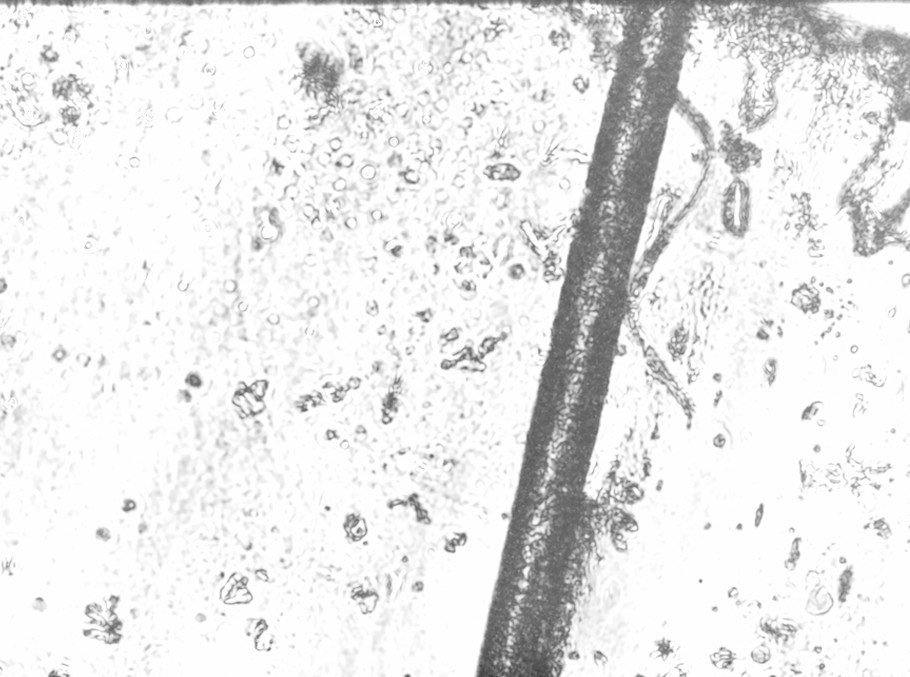
Example image of a human hair captured by the holographic microscope following manual image reconstruction The prickly background is caused by the sample preparation with a transparent adhesive tape (background noise of the image)

The image in figure 6 (Fig. 6) shows a human hair captured with the student-build microscope. Holographaphic microscopy provides topographical information of the sample that cannot be attained using conventional transmission microscopy. The system records interference patterns of a scattered laser beam, from which both amplitude and phase information of the sample are computationally reconstructed using digital holography techniques. As the used cost effective single board computer (a raspberry pi 4B with 4 gigabytes of RAM) does not provide the necessary computing power, the capturing process was separated from the visualization and display process. 

The captured file needed complex image reconstruction (with a runtime of about 2 minutes to compute the image), that have to be run separately, leading to an high-resolution pseudo-3D image, despite of the minimal material cost (under 200 €). 

## Discussion

Participation in the project was notably higher compared to other non-mandatory classes within the course, which typically have an average attendance rate of 30-40%. This high level of engagement reflects the project's perceived relevance to the students' educational goals. Throughout the project, students encountered challenges, including the availability of specific hardware components and issues related to group dynamics. These challenges align with prior research on interdisciplinary collaboration and practical problem-solving skills [1]. In cases where electronic components were unavailable, students adapted by selecting alternative parts, requiring them to learn additional skills such as soldering and electronic integration. This supported the development of higher-order thinking and problem-solving skills, as intended by Bloom’s Taxonomy [7]. These adaptations highlighted the importance of flexibility and problem-solving in engineering projects. Organizational challenges also arose as students navigated teamwork dynamics, which highlighted the need for targeted support in managing communication and group coordination. These experiences provided significant learning opportunities, connecting to situated learning theory by exposing students to real-world technical and organizational obstacles commonly encountered in professional settings [5]. The educational outcome aligns with socio-constructivist principles, emphasizing active and collaborative learning. Students took ownership of their projects, a factor that likely contributed to the observed improvements in motivation and exam performance. The use of open-source designs motivated students to contribute back to the academic community by sharing improvements and modifications, fostering an appreciation for scientific knowledge-sharing. Despite its success, the project faced some challenges. The non-mandatory nature of the course introduced self-selection bias, which may have influenced the results. Additionally, logistical issues, such as delays in component delivery and varying levels of team collaboration, required flexibility and problem-solving. These limitations underscore the importance of structured guidance and ongoing support in project-based learning. Future iterations of the project will focus on refining the curriculum to better support teamwork and exploring additional applications of rapid prototyping and cross-disciplinarity in biomedical education. Expanding the use of open-source designs and improving the integration of evaluation metrics, such as detailed statistical analyses and peer-reviewed assessments, will further enhance the program’s effectiveness. Additionally, collaborations with medical faculties could provide a pathway for broader implementation of this educational model. Furthermore, the project provides a foundation for interprofessional education, where medical and engineering students could collaborate on solving real-world problems, fostering early teamwork skills akin to clinical scientist programs.

## Conclusion

The project highlights the importance of cross-course and interdisciplinary learning, as students applied their technical knowledge in medical contexts, bridging the gap between engineering and healthcare and demonstrated the value of cross-disciplinary project based learning in combination with rapid prototyping and open-source technology in medical education. By engaging students in building their own microscopes, this project promoted scientific collaboration and technical expertise, equipping future biomedical engineers with the skills necessary for their professional roles. While the project was designed for biomedical engineering students, its core principles are transferable to medical education. The integration of open-source tools and hands-on methods enhanced anatomy and physiology courses, making complex concepts more accessible and engaging. This could also be applicable for medical or nursing students. The combination of theoretical lectures, interactive seminars, and hands-on labs provided a comprehensive learning experience, preparing students for real-world biomedical challenges. The project also demonstrated that DIY and open-source approaches can offer sustainable, cost-effective alternatives to traditional lab equipment. 

## Authors’ ORCIDs

- Christian Hanshans: [0000-0002-1923-7791]
- Friederike Burkhardt: [0009-0000-4306-7051]

## Competing interests

The authors declare that they have no competing interests.
